# An extrapolation of Foucault’s *Technologies of the Self* to effect positive transformation in the intensivist as teacher and mentor

**DOI:** 10.1186/1747-5341-8-7

**Published:** 2013-07-18

**Authors:** Thomas J Papadimos, Joanna E Manos, Stuart J Murray

**Affiliations:** 1 Department of Anesthesiology, The Ohio State University, Wexner Medical Center, Columbus, OH, USA; 2 Department of Integrated Studies, Southern New Hampshire University, Manchester, NH, USA; 3 Department of English Language and Literature, Carleton University, Ottawa, Ontario, Canada

**Keywords:** Mentoring, Medical education, Critical care, Philosophy, Teaching

## Abstract

In critical care medicine, teaching and mentoring practices are extremely important in regard to attracting and retaining young trainees and faculty in this important subspecialty that has a scarcity of needed personnel in the USA. To this end, we argue that Foucault’s *Technologies of the Self* is critical background reading when endeavoring to effect the positive transformation of faculty into effective teachers and mentors.

## Introduction

Critical care medicine is demanding, regardless of whether the intensivist is an anesthesiologist, surgeon, or internist. The demand for critical care services and expertise is growing worldwide [1]. With our training structures being regarded as inadequate and with the worsening shortage of available critical care physicians we face a difficult situation [2]. The workload is increasing dramatically [3], and accordingly, the “burnout” rate in the field is becoming of great concern [4]. So while this commentary could apply to any physician educator/mentor, we discuss intensivists in particular because of the acute stress and introspection required in their daily routines, especially given their entanglements in end-of-life matters and the complexity of their care delivery model. Specifically, depression and burnout among intensivists is very high [5-7]. The costs of educating such a highly skilled workforce, and then replacing it before the end of its anticipated life expectancy, has enormous medical, social, and economic costs [8]. Effective mentoring, role modeling, and self-examination are integral to having an adequate and successful critical care workforce in the future as our aging population grows [9].

Additionally, the practice of critical care now faces serious economic constraints, growing government oversight, and a dramatically aging society coupled with new medical technologies, such as genome mapping and life-extending medical devices, procedures, and pharmaceuticals [10-12]. To attract our young medical school graduates to this strenuous subspecialty it is of paramount importance that those of us who practice in this field become good role models, but beyond this we must also become good teachers and mentors. Here we argue that Foucault’s *Technologies of the Self* is critical background reading when endeavoring to effect the positive transformation of faculty into effective teachers and mentors in our training programs, both at the undergraduate and graduate levels. Foucault’s thoughts applied through formal and informal mentoring programs can position mentors/teachers and trainees to better address these challenges and to improve their skills through transformation of self.

## Discussion

Being a teacher and mentor in medicine is an ethical calling that demands transformation, not only concerning what one knows, but in relation to what one is, and what it is good to *be*. Faculty members should be teachers and mentors who continue to grow intellectually, socially, and spiritually over time. And this involves a devout commitment to educating the next generation of physicians. Foucault states that, “a certain structure of spirituality tries to link knowledge, the activity of knowing, and the conditions and the effects of this activity, to a transformation in the subject’s being” [13]. He further comments that, “The main interest in life and work is to become someone else that you were not in the beginning” [14]. This is an interesting and challenging concept, this idea of self-*transformation*, whereby “there cannot be knowledge without a profound modification in the subject’s being” [13]. An insightful and excavative comment with which Michel Foucault, a distinguished philosopher, historian, poststructuralist, and in no small way a teacher of note, teases us. This is a self-analytical socio-archaeological excavation that, at first blush, seems to be a reflective action that an individual (in this dialectic, a teacher and/or mentor) may wish to pursue only in the autumn of a career. However, such reflection is not meant to occur as a terminal singularity, at the end of a life lived. It is meant to be an ongoing engagement in the “historical reflection of our self” [15], i.e., a continuing reflective process throughout a life, or career. Teachers/mentors should ask themselves frequently, as Kant did in an essay that reflects on the Enlightenment, “what are we in our actuality, what are we today?” [15]. In effect, we need to ask ourselves, “How does a human being turn him- or herself into a subject?” [15]. In other words, what are the normative terms in and through which we are subjectivated, in and through which we understand ourselves as social and ethical creatures in relation to others? To whom do these terms belong? And how should they define us? This interrogation is profoundly important in that it strikes at the heart of growing as teacher and mentor; it is the basis of self-transformation vis-à-vis others.

The technology of the self is an investigative and collaborative process whereby teachers and mentors, by their own means or with the help of others, act upon themselves to change their thoughts, conducts, and way of being or teaching. This process is necessary as it leads to self-transformation and the attainment of a more “enlightened” state in order to become a sage or even an immortal in word or deed. In other words, the technology of the self is the history of how an individual acts upon himself or herself [16]. Foucault states that this endeavor “is worthwhile insofar as we don’t know what will be the end” [17]. This attempt at the introspection of the self as subject becomes an imperative and on-going action, the means by which we transform ourselves as teachers and mentors through reflection over a career.

So, how do we reflect? No small task. Being honest with one’s self through reflection can be difficult. Who I am really lies somewhere between who I think am, who others think I am, and how this is communicated. While Foucault’s thoughts and writings on the technologies of the self were not fully developed at the time of his premature death in 1984, he did leave us his initial perspectives on the topic [18]. We would like to develop his initial approach to the technologies of the self, specifically from three perspectives: (1) the view of the ancients regarding “concern for one’s self” and “knowing oneself,” (2) the relation of caring for the self and defective education, and (3) the relation between care for oneself and care for the political life (taken by Foucault primarily from Plato’s *Alcibiades*). We will read these with a view to the transformation of faculty members in their role as teachers and mentors.

Foucault tells us that in order to develop ourselves, we must be concerned with ourselves. However, “concern,” historically, came to be replaced with “knowing one’s self.” Is there a difference? Yes there is a difference. To use a historical example, the oracle at Delphi insisted that any supplicant to the gods should “know themselves” because if you know yourself you will take care as to what you ask for, and at the same time, understand the ramifications of any advice that is given to you. Knowing yourself, in ancient times, was associated with being concerned with yourself, thereby creating an initial requirement to occupy one’s self with one’s self. The latter needs to come into play before the former.

Therefore, it is extremely important for teachers and mentors to occupy themselves with themselves, since this will lead to introspection regarding their academic advancement, a frank awareness of their individual limits, and an understanding first of what their knowledge is *for*, what ends it will serve. In this regard, they should be concerned about their teaching abilities, their record of publications, service to their institution, their place within their department and institution (with respect to policies, procedures, and relationships), and their ability to project a regional, national, or international reputation. When faculty members begin to occupy themselves with themselves (a true concern about their abilities and advancement), then they will occupy themselves with concerns regarding the greater institution and its purpose, much in the way that Socrates claimed that in teaching people to occupy themselves with themselves they will also occupy themselves with the greater good of their city [14]. If you are occupied with the true pursuit of excellence, then you and your institution and your profession benefit.

However important it may be for teachers and mentors to begin this process of self-reflection by occupying themselves with themselves in order to benefit professionally, Foucault’s message is, in fact, much more radical. Citing Socrates, Foucault says that care of the self is distinctly not care for one’s possessions or one’s reputation. It is care for one’s soul, and the soul’s relation with itself is a matter of *khresis* or “use” – but absolutely not “use” in any instrumental sense. He says explicitly when the doctor is sick and prescribes medication to himself, he is not caring for himself in the sense that Socrates intends. Care of the self is spiritual.

It is so unbelievably radical because it overturns the foundations of Western reason and what it means to have “access” to the truth. Since the Cartesian moment, Foucault says, modern subjects believe they have access to the truth by virtue of a cognitive relation (e.g., one has correct mental representations that correspond to the outside world). But the care of the self turns things upside down: the ancient sense (carried down through some religious traditions) is that one must have the kind of spiritual relationship with oneself that will prepare the way for the reception of truth. Cognitive relationships are not privileged, as they are in modernity; rather, spiritual relationships are.

At this juncture we must also acknowledge that while it is important to know oneself and care for oneself, it is equally important to know “thy limits.” The concept of limits is important for an educator. The teacher/mentor must recognize the limits of self-reflection, the limits of one’s capabilities and knowledge, and to communicate this intellectual honesty to students. Such an admission and demonstration of “limits” is important to mentorship and professionalism. In other words, finding a way to mentor the mentors is important [19].

In the context of critical care medicine, how does a teacher/mentor begin to reflect in this manner? We must first make several assumptions regarding the institution with which the teacher/mentor is affiliated. The first assumption is that the institution will only hire physicians who wish to teach. The candidate must enjoy teaching and have demonstrated scholarly activity, or some attempt at scholarly activity, and will have demonstrated an inclination to teach. The second assumption is that the institution will have mechanisms, policies, and curricula in place to assist the teacher/mentor who is committed to his or her own improvement over time, or *transformation*. Thus, the institution does not seek a deficient subject for incorporation into its “polis.” If the institution only hires faculty with the minimal qualifications required in order to fill a full-time equivalent position so as to get the clinical job done, then there will be a deficiency in the talent needed for institutional academic excellence. While it would seem easy to avoid this dilemma by merely having a particular portion of the department assigned to teaching, or to have those with the requisite interest and teaching/mentoring skills to spend proportionally more time with trainees, this would preclude the ideal mentor who is teacher/researcher/clinician faculty from the institutional mix. Instead of an institution looking for this ideal faculty member, the administration would be tempted to compartmentalize educational “streams” to minimize costs. These are the individuals who departments and institutions should strive to acquire as faculty members. In other words, they ought to seek teachers who will be good mentors and in whom the spirituality of education and mentoring is present. As previously mentioned, the ancients believed that this spirituality was a condition of access to truth [13]. Such a spiritual relationship might facilitate – or provide the condition for – a certain “access” to the truths of medical research and clinical practice. After all, the relationship of care is the basis of medicine.

Currently, some universities seem to want to take a pragmatic perspective to the issue of reflective thought regarding the advancement of teaching and mentoring by advocating the following: (1) a program of peer review and mentoring of lectures and teaching methods (at the bedside and otherwise) should be a departmental policy for advancement in rank, (2) teaching simulators, as opposed to strictly clinical simulators, should be developed in which skills can be developed and assessed, (3) video recordings of teaching encounters with review by the teacher and their mentor, (4) evaluations by trainees of their teachers (which is required by most accrediting agencies), (5) assignment of mentors for all faculty, and (6) monetary consequences/incentivizing for deficient performance can, at times, cause considerable self-reflection. “Caring” for one’s self in such a manner may well limit some deficiencies in teaching and mentoring, and by default, some would say, in this manner it is possible for effective reflection to occur.

However, a Foucauldian perspective would yield dramatically different results. None of the above “pragmatism” would sit well with him because none of these pragmatic solutions would help you to “know yourself” (*gnothi seauton*), an important concept in the transformation/transfiguration of a teacher/mentor. And as Foucault reminds us, *gnothi seauton* must be preceded by the spiritual relation, *epimeleia heautou* (to care for one’s self). There are several general notions to bear in mind with *epimeleia heautou*. The first is that the teacher/mentor must take “care of oneself, attending to oneself, being concerned about oneself, etcetera” [17]. Next, teachers/mentors cannot know themselves unless they have cared for themselves. This means paying attention to oneself, but it is more serious than this. It means reckoning seriously what you own and for those things for which you are responsible and for your health; such activities were viewed as political by the Greeks, and of utmost necessity. Second, *epimeleia heautou* also has the implication of an inward look: as teachers/mentors “we must convert our looking from the outside, from others and the world, etc., toward ‘oneself’” [17]. We need to be aware of what we think and what occurs during our exercise of thought. Thirdly, *epimeleia* involves exercises of the mind, such as “techniques of meditation, memorization of the past, of examination of conscience, of checking representations which appear in the mind” [17]. In other words, these are “technologies,” actions that we must take upon ourselves by ourselves that will allow us to take responsibility for who we are and what we do. These techniques are the means by which “one changes, purifies, transforms, and transfigures oneself” [17]. As is evident from the aforementioned discussion, Foucault could not abide by the pragmatic approach to reflection.

Care of the political life is another crucial component in the academic’s life as a teacher and mentor. Foucault, through Plato’s writings, tells us that part of the concern for one’s self always refers to an active political state. This activity is something in which the teacher/mentor actively engages. It is not just a perspective or attitude. The ancients tended to their fields, their cattle, their homes, jobs, the city, and their citizens-colleagues. In the same way, an effective teacher/mentor must tend to their scholarly activity, their department, engage in appropriate hospital/college committees, support their colleagues, and the university/teaching setting. If a teacher/mentor wishes to gain advantage s/he must acquire *technai*, i.e., the skills needed for survival and advancement in order to gain the upper hand (in the setting that we are addressing). Understanding political surroundings and the implications of the immediate academic environment will help in the transformation of the teacher/mentor by making him/her more effective. Foucault would add, “think about who you actually are, check your strengths and weaknesses, think about who you are in comparison with those you want to confront, and you will discover your inferiority” [13]. Mentors must pay heed to themselves and apply their minds to their self, with as full an awareness as possible of their weaknesses and limitations [13].

This perspective regarding introspection/reflection as laid out by Foucault in *Technologies of the Self* is more than theoretical fancy when we consider mentoring in medicine. To begin with, there are very “few programs that exist to both improve the effectiveness of established mentors and cultivate a mentoring community” [20]. In fact, a systematic review of mentoring in academic medicine settings by Sambunjak et al. found that there was a need for more rigorous methods of establishing evidence to support the perception that mentoring was important [21].

While extolling the need for the incorporation of the theoretical aspects of *The Technologies of the Self* in the foregoing dialectic, one may ask for more specific examples of how this may be accomplished or applied**.** Below we present the demonstration of three specific examples of the Foucauldian perspective and their application to the education of mentors (and their mentees).

The first is the use of narrative therapy, or writing narratives. Michael White, a key figure of the narrative movement of the 1990s, was a family therapist who actively drew on Foucault as a technique. He helped patients by allowing them to externalize their problems:

“White regards the process of externalizing as a counter practice to cultural practices which objectify persons and their bodies. It enables people to separate from the dominant stories that have been shaping their lives and relationships, and it opens spaces to re-author themselves. He avoids individualizing the problem, while retaining the notion of responsibility through improving the capacity for personal agency in the pursuit of new possibilities” [22].

This technique of narrative therapy, based on the work of Foucault (using a postmodern analysis – see example no. 3 that follows) can be used to help mentors, and, in turn, for mentors to help their mentees regarding difficulties faced with research, collaboration, and negotiations regarding time and money, etc. [23,24].

Second, the traditional dyadic methodology seems to be falling by the wayside while informal mentoring relationships that are formed through common interests between mentor and mentee seem to be more effective, lasting, and successful [25,26]. Nonetheless, informal relationships are still much less common than the dyadic paradigm.

**Figure 1:**
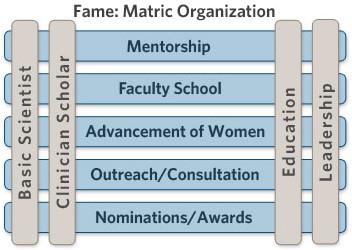
The Ohio State University FAME matrices.

To replace this more traditional approach, mentoring networks and programs to “mentor the mentor” are being formed. At The Ohio State University College of Medicine the academic promotion process has become very challenging. Candidates for promotion to all faculty ranks need evidence of outstanding teaching scores, evidence of peer-reviewed teaching, evidence of being a successful mentor, evidence of scholarly work of significance, service to their department and institution, and a national reputation (for Associate Professor) and national leadership (for Professor). To assist in self-assessment and preparation for advancement in rank the medical center and medical school have created The Center for FAME (Faculty Advancement, Mentoring and Engagement). FAME is a comprehensive multi-faceted faculty development office. It is designed to synergize existing resources and provide programming to support each faculty member’s career development. FAME has identified four primary pillars (see Figure 1) wherein a faculty member will realize opportunities for career growth and development. Across these four pillars are the programs that support the faculty member’s development. However, FAME also deals with mentoring the mentors and improving their effectiveness and outreach to students, residents, and other faculty mentors. In fact, mentoring mentors and mentoring mentees in how to deal with the high level of rejection in academic medicine and the need for resilience is of paramount importance [27].

In these new programs and in centers such as FAME faculty members are not only told what milestones are important for success, but are also given the necessary tools to assess themselves. Essentially, they are instructed in the *tekne* of self to make themselves better teachers, role models, mentors, and yes, scholars.

An additional example concerning mentoring relationships is found in The Faculty Mentoring Leadership Program (FMLP) at Brigham and Women’s Hospital (BWH). LC Tsen et al. recently described the BWH’s rationale, design, implementation, evaluation, and on-going impact of their program [20]. The FMLP “was created to enhance the mentoring skills of midcareer and senior faculty physician and scientist mentors” [20].

Foucault’s work helps support the paradigm of the use of mentor networks, as opposed to the traditional dyadic model. The subject (the mentor) will find better support through a network of mentors because each can provide a different perspective on a singular topic, or collectively they will the subject, or council the subject, in light of the different discourses that engulf/influence the subject. In light of Foucault’s postmodern critique, these networks are:

“Valuable in drawing attention to the way in which selves can participate in their own subjugation, and how existing adult education technologies, in the name of promoting autonomy and freedom, can be accomplices in the process of subjugation. As such it is important to ‘deconstruct’ such technologies and the selves produced by them. But, what of the reconstruction of selves? In this regard, the postmodern focus on the self as text offers new possibilities for self work” [22].

Third, it is useful to consider adult education as belonging to and extending this leverage of the technologies of self in the academic world of the intensivist, and other specialties, in that having the liberty to do and say things is of great importance, i.e., academic freedom. Foucault’s work and viewpoints are important here:

“Foucault, for one, regarded a permanent critique of ourselves, and the relation of self to self, as central to the practice of liberty. Perhaps the most powerful notion here is the situated self: the self as part of the text of the world, which opens up the possibility of refusing the way one has been inscribed and exploring alternative discourses about one’s self as a means of resisting domination and oppression” [22].

Whether facing other faculty members, administrators, or students, “the importance of developing a concept of the self which is compatible with the transformative (and thereby resistant) adult education practices is without question” [22]. Foucault has been involved or associated with the development of a way of viewing subjectivity as not being reliant on the individual-society dualism of the ‘modern’ viewpoint:

“It does so by reconceptualizing and renaming the terms of dualism, so that ‘individual’ and ‘society’ are replaced by the concepts of ‘the subject’ and ‘the social’, which are understood as produced rather than as pre-given and then interacting. Thus post-modernism problematizes at the outset the concepts of individual and society as effects which are produced rather than as pre-given entities” [22].

So in the Foucauldian view the individual is replaced by the idea of the subject (mentor or teacher), a subject within a particular discourse. New deans, new administrators, and department chairs come and go frequently in an institution, along with various political dialogues about research, parking garages, teaching, book stores, events on campus, etc. The idea espoused by Foucault is that because of the diversity of discourses, there may be a number of possible subject positions that an individual may be forced, or may wish, to occupy. Therefore, it is important to teach mentors that multiple subject positions within multiple discourses might be acceptable and could be powerfully liberating. Specifically, you could teach faculty that they may be deconstructed historical products, the effects of implanted discourses in the daily practices of the university [22]. This perspective can be supported by university centers such as FAME and FMLP.

The three above-mentioned specifics dovetail very well with the new concepts/viewpoints on mentoring that have been espoused in recent literature and cited in our arguments. The ideas of narrative therapy/exercises, informal mentoring/mentoring networks, and organized centers for mentoring within universities are powerful concrete educational policy supplements to a new practical framework for mentoring.

Our focus to this point has been on the educator/mentor and the concept of the technologies of the self from the Foucauldian perspective. However, while self-insight, knowledge, and the political climate surrounding the physician in academic medicine are of great importance, we must not lose sight of those who we are charged to educate – our medical students. These trainees are not just our moral responsibility; they are the future of the medical profession. While we need to reflect more effectively, be appropriately introspective, care for ourselves, and know ourselves better, in the end the bottom line is that we must lead. These exercises may be helpful to our transformation as teachers/mentors, but the injunction to improve our selves must help us to carry on our important work in regard to the guidance, care, tutelage, and instillation of a moral conscience in our students in order to make them effective healers and purveyors of truth. Our ability to be admired role models and to successfully mentor our juniors will medically, socially, economically, and politically affect our patients and society in general. The teaching of therapeutic and moral competence is the goal of a medical educator and the teknai we allude to in this commentary can provide a philosophy of reflection/introspection and self-evaluation that is transformative and of distinctive merit to the educator and those they mentor. To prepare those who follow us, we must at once be the student and the teacher [28].

Michel Foucault’s Technologies of the Self is an outstanding philosophical supplement to introspection and reflection for the mentor/teacher. Although other philosophers’ instruction could be equally helpful, here we have argued on behalf of Foucault’s insights and thoughts. This is germane, too, in regard to the variety of roles that mentors must play, including, “being a professional parent, teacher, guide, counselor, motivator, sponsor, coach, advisor, role model, referral agent and door opener” [29].

## Conclusion

Technologies of the self is not an “activity.” It is a set of practices that can assist in the transformation of one’s self over time. These practices may be competing and cause moral conflict. It is the care the self that is, essentially, a transformative practice requiring knowledge of one’s self, education of one’s self, and understanding of the political discourses and stakeholders that encompass the mentor’s teaching environment, especially in critical care medicine. While such activity most importantly incorporates reflection, it is also requires vigilance – vigilance of one’s self and one’s environment. As Socrates told Alcibiades, “pay some attention, reflect a bit on what you are, look at the education you have received, you will do well to know yourself a little better” [30]. This is especially true at this point in time when our aging and rapidly advancing technological society is exposed to a polarized and mercurial politico-economic climate.

## Competing interests

The authors declare that they have no competing interests.

## Authors’ contributions

TJP conceived, researched, wrote, and edited the manuscript. JEM wrote and edited the manuscript. SJM researched, wrote, and edited the manuscript. All authors read and approved the final manuscript.
